# When will the Glomerular Filtration Rate in Former Preterm Neonates Catch up with Their Term Peers?

**DOI:** 10.1007/s11095-024-03677-3

**Published:** 2024-03-12

**Authors:** Yunjiao Wu, Karel Allegaert, Robert B. Flint, Sebastiaan C. Goulooze, Pyry A. J. Välitalo, Matthijs de Hoog, Hussain Mulla, Catherine M. T. Sherwin, Sinno H. P. Simons, Elke H. J. Krekels, Catherijne A. J. Knibbe, Swantje Völler

**Affiliations:** 1 Division of Systems Pharmacology and Pharmacy, Leiden Academic Centre for Drug Research, Leiden University, 2333CC Leiden, The Netherlands; 2 Department of Hospital Pharmacy, Erasmus University Medical Center, Rotterdam, The Netherlands; 3 Department of Development and Regeneration, and Department of Pharmaceutical and Pharmacological Sciences, KU Leuven, Leuven, Belgium; 4 Department of Pediatrics, Division of Neonatology, Erasmus MC Sophia Children’s Hospital, Rotterdam, The Netherlands; 5 Leiden Experts On Advanced Pharmacokinetics and Pharmacodynamics (LAP&P), Leiden, The Netherlands; 6 School of Pharmacy, University of Eastern Finland, Yliopistonranta 1 C, 70210 Kuopio, Finland; 7 Finnish Medicines Agency, Hallituskatu 12-14, 70100 Kuopio, Finland; 8 Department of Neonatal and Pediatric Intensive Care, Division of Pediatric Intensive Care, Erasmus MC Sophia Children’s Hospital, Rotterdam, The Netherlands; 9 Department of Pharmacy, University Hospitals of Leicester, Glenfield Hospital, Leicester, LE39QP England; 10 Department of Pediatrics, Wright State University Boonshoft School of Medicine/Dayton Children’s Hospital, One Children’s Plaza, Dayton, OH USA; 11 Certara Inc, Princeton, NJ USA; 12 Department of Clinical Pharmacy, St Antonius Hospital, Nieuwegein, The Netherlands

**Keywords:** creatinine, glomerular filtration rate, inulin clearance, maturation, preterm neonates

## Abstract

**Aims:**

Whether and when glomerular filtration rate (GFR) in preterms catches up with term peers is unknown. This study aims to develop a GFR maturation model for (pre)term-born individuals from birth to 18 years of age. Secondarily, the function is applied to data of different renally excreted drugs.

**Methods:**

We combined published inulin clearance values and serum creatinine (Scr) concentrations in (pre)term born individuals throughout childhood. Inulin clearance was assumed to be equal to GFR, and Scr to reflect creatinine synthesis rate/GFR. We developed a GFR function consisting of GFR_birth_ (GFR at birth), and an Emax model dependent on PNA (with GFR_max_, PNA_50_ (PNA at which half of ${GFR}_{max}$ is reached) and Hill coefficient). The final GFR model was applied to predict gentamicin, tobramycin and vancomycin concentrations.

**Result:**

In the GFR model, GFR_birth_ varied with birthweight linearly while in the PNA-based Emax equation, GA was the best covariate for PNA_50_, and current weight for GFR_max_. The final model showed that for a child born at 26 weeks GA, absolute GFR is 18%, 63%, 80%, 92% and 96% of the GFR of a child born at 40 weeks GA at 1 month, 6 months, 1 year, 3 years and 12 years, respectively. PopPK models with the GFR maturation equations predicted concentrations of renally cleared antibiotics across (pre)term-born neonates until 18 years well.

**Conclusions:**

GFR of preterm individuals catches up with term peers at around three years of age, implying reduced dosages of renally cleared drugs should be considered below this age.

**Supplementary Information:**

The online version contains supplementary material available at 10.1007/s11095-024-03677-3.

## Introduction

Preterm neonates, defined as neonates born before 37 weeks of gestation, display immature organ function and, therefore, typically require lower drug doses compared to their term peers [1, 2]. However, from what age prematurely born individuals can be treated with the same drug doses as their term peers has not been systematically studied before. Closing this knowledge gap requires understanding the maturation of key physiological processes that drive pharmacokinetics [3–5]. One of those key physiological processes is the renal function. Renal elimination is influenced by glomerular filtration, tubular secretion, and tubular reabsorption, with glomerular filtration being the most relevant process [6] and the focus of the current study.

In normal pregnancies, nephrogenesis starts from the 6th week of gestation and is typically completed by 36 weeks of gestation [7]. Preterm birth has been associated with a reduced nephron number [8, 9], smaller kidney volumes, and a higher percentage of morphologically abnormal nephrons [10, 11]. In addition, preterm neonates are exposed to nephrotoxic drugs and experience hypotension and sepsis more frequently, which can further limit their renal function. Consequently, preterms have a reduced GFR at birth, coupled with a slower postnatal GFR development [12, 13]. Until now, it remains unclear whether and when the GFR of prematurely born individuals catches up with their term counterparts [13–21]. This uncertainty primarily arises from the absence of GFR measurements across the entire age range from preterm-born individuals.

GFR can be assessed using endogenous or exogenous markers. Among them, inulin clearance (CL) is a direct marker and is considered the gold standard. However, its measurement is associated with a considerable practical burden on both practitioner and patient, *i.e.,* a required inulin infusion followed by timed plasma and urine collection, preventing its wide accessibility [22, 23]. Serum creatinine (Scr), on the contrary, is an indirect measure of GFR with less accuracy and is commonly used to calculate the estimated GFR (eGFR). Creatinine is the waste product of muscle tissue and is released into the blood at a specific rate, *i.e.,* synthesis rate, after which it is eliminated from the blood. Elimination occurs primarily through glomerular filtration, with only minor contributions of tubular secretion and reabsorption processes [24]. For neonates, the Scr concentration in the first few days of life is also impacted by the maternal concentrations [25]. Many Scr-based equations have been developed to calculate eGFR in children, including the Schwartz-equation [26, 27] and its derivations. These equations mainly differ in describing the synthesis rate of creatinine, which frequently considers factors such as body size, occasionally in conjunction with variables like age, sex, and specific medical conditions [28–36]. Other equations estimate GFR by comparing the individual Scr concentration with reference values [37, 38]. Few have been developed for neonates, and even fewer for preterm neonates [26, 27, 33, 34].

This study aims to develop a mathematical model to describe the maturation of GFR from birth to 18 years of age for individuals varying in gestational age (GA) between 24–44 weeks, ultimately to determine whether and when the GFR of preterm-born individuals catches up with their term peers. To this end, inulin CL data from term and preterm-born individuals were combined with Scr concentrations for model development. The developed GFR maturation equation was subsequently explored as a covariate function of CL in popPK models of three renally cleared drugs for its predictive performance for gentamicin, tobramycin, and vancomycin concentrations across the entire age range from preterm and term neonates to 18 years of age.

## Methods

### Dataset

The GFR maturation model was informed by pooling inulin CL values collected from literature [12, 39–42] and Scr concentrations originating from four clinical trials. More details are given below.

#### Collection of Inulin Clearance Values

Individual inulin CL values from both preterm and term-born infants (333 subjects) under 90 days of PNA were collected from a previous publication [12]. Since the aforementioned publication [12] exclusively covered inulin CL data up to 90 days of age, additional inulin CL data from 15 subjects exceeding 90 days of PNA were extracted from two studies [43, 44] that were part of the previous paper. More details on the search strategy and inclusion criteria can be found in the original publication [12]. An additional literature search to identify papers in children above 90 days of age was conducted on January 23rd, 2023, via *Pubmed*. Papers meeting the following criteria were included: provides individual inulin CL data along with age and at least one of the following demographic information: current weight, height, or body surface area (BSA). Based on this search, four additional papers [39–42]with data from a total of 35 subjects aged above 1.6 years were included. In total, inulin CL data from 50 subjects were added to the previous number of 333 subjects from the previous study [12], resulting in inulin CL data from 383 subjects.

#### Collection of Scr Concentrations

- **Dataset 1** The dataset included 71 preterm and term-born patients, ranging from birth to 17.7 years of age, who were admitted to ICUs at the Sophia Children's Hospital between June 1st, 2017, and February 5th, 2021. This study was approved by the Medical Ethical Review Committee Erasmus MC (MEC-2023–0647) which waived the need for informed consent. Creatinine concentrations of the venous blood samples were performed at the laboratory of the clinical chemistry department at Erasmus MC. Creatinine plasma concentrations were measured by an enzymatic immunoassay (Cobas8000 system, Roche Diagnostics, Basel, Switzerland).
- **Dataset 2** The dataset included 98 preterm neonates born before 32 weeks GA and aged under 229 days of PNA, who were recruited in the Drug dosage Improvement in Neonates (DINO) study [45–47] (NL47409.078.14, MEC-2014–067, NCT02421068) between September 2014 and July 2017. Creatinine concentrations of the venous blood samples were performed at the laboratory of the clinical chemistry department at Erasmus MC. Creatinine plasma concentrations were measured by an enzymatic immunoassay (Cobas8000 system, Roche Diagnostics, Basel, Switzerland).
- **Dataset 3** This dataset contained 125 subjects of around 11 years of age (NCT02147457), including prematurely born individuals who were born between 2000 and 2005 in the University Hospital Leuven (Belgium) with a birth weight < 1,000 g and term controls that were either friends of the included cases or recruited at an elementary school close to the examination center in Eksel, Belgium [48]. Scr was measured by the enzymatic Creatinine Plus Generation 2 kit, running on a COBAS Integra 400 system (Roche Diagnostics, Basel, Switzerland). Measurements of Scr was calibrated by isotope-dilution mass spectrometry [48].
- **Dataset 4** This dataset contained 100 subjects of around 11 years of age, including prematurely born individuals with birthweight < 1,000 g recruited from the outpatient paediatric department of the Polish American Children’s Hospital in Krakow and term neonates with birthweight of > 2,500 g who were recruited from a general practitioner’s office [17]. Scr concentrations were measured using VITROS® Chemistry Products (Ortho Clinical Diagnostics, Raritan, NJ).

#### Exclusion of Scr During Acute Kidney Injury (AKI)

For dataset 1 and dataset 2, Scr concentrations during AKI, defined as Scr increase ≥ 0.3 mg/dL or Scr increase ≥ 150% of the previous observation [49], were not included in the dataset. For subjects with PNA below 1 year, the exclusion continued until the concentrations returned to a value equal to or below the last recorded Scr concentration defined as normal. For PNA above 1 year, concentrations were excluded until the concentrations returned to a value equal to or below 150% of the last normal Scr value or 0.3 mg/dL plus the last normal Scr concentration. The cut-off value of 1 year for the different criteria was selected because Scr concentrations generally decrease with PNA below 1 year of age and then increase after that [50]. As we could not link neonatal AKI to subsequent renal function [51, 52], we chose not to exclude the later Scr concentrations from patients (n = 22) with previously defined AKI from our study.

#### Imputation of Missing Demographic Information

Of the 50 newly included subjects in the inulin CL dataset, thirteen subjects from the study of Brodehl *et al. *[43] were reported as term-born and aged above 2 years, except for one who was 120 days old. All these subjects were assumed to have a GA of 40 weeks with a median birthweight of this GA population based on the Fenton growth chart [53]. Two subjects from Barnett *et al. *[44] had birthweight reported, and therefore, GA was calculated assuming the birthweight was the median of the corresponding GA [53]. For the other 35 subjects [39–42] above 1.6 years, both information on GA and birthweight were missing. In this case, GA was assumed to be 40 weeks, and birthweight was calculated accordingly [53]. Missing sex (n = 7) was imputed by random generation from a binomial distribution with a probability of 0.5. At least current weight or BSA values were reported for all these inulin CL values. In case one of the two was missing, the missing value was derived from the available value and the height of the individuals using the Haycock equation [54]. If height was also missing, the missing height was generated using the standard height curves (details in supplementary materials).

For all Scr values, individual information on GA and birthweight was available. When the current weight was missing within a subject with multiple weight measurements, linear interpolation was used to obtain the missing values. For missing weight values that could not be interpolated, if the time gap between the nearest weight observation and the missing observation was shorter than 10 days, the nearest observation was extrapolated. Otherwise, the missing values were predicted based on the trend of the standard weight growth chart (details in supplementary materials). The imputation of missing height is similar to the current weight; only the maximum time for extrapolation was extended to 60 days. The Haycock equation calculated BSA using current weight and height [54]. Each patient's fat-free mass (FFM) was calculated using the function by O’Hanlon *et al. *[55].

### Model Development

The model development was performed in NONMEM V7.4.3 (ICON Development Solutions, Ellicott City, MD, USA), supported by Perl-speaks-NONMEM (PsN) 4.9.0, and interfaced by Pirana 2.9.9 (Certara). Processing and visualization of output from NONMEM were performed in R 4.2.3 (CRAN.R-project.org).

#### Base Model

A GFR maturation model that simultaneously characterizes inulin CL values and Scr concentrations was developed. Because it was assumed that inulin CL is the gold standard method for GFR determination, inulin CL was set to be equal to GFR (Eq. 1). Creatinine concentrations were assumed to be at a steady state so that the concentrations could be determined by the synthesis rate (Syn) and GFR (Eq. 2).

1 $$Inulin\;CL=GFR$$

2 $$Scr=\frac{Syn}{GFR}$$

The maturation of GFR was assumed to have distinct prenatal and postnatal patterns, as found in our previous study [12]. Prenatal maturation is reflected in GFR at birth (${GFR}_{birth}$), for which a linear function with birthweight was applied, according to Wu *et al*. [12]. For postnatal maturation, a sigmoidal function based on PNA, as described in the literature [56] was used:

3 $${GFR}_{birth}={TVGFR}_{birth}\times (\frac{birthweight}{1750})$$

4 $$GFR={GFR}_{birth}+\frac{({GFR}_{max}{-{GFR}_{birth})\times PNA}^{\gamma }}{{{PNA}_{50}}^{\gamma }+ {PNA}^{\gamma }}$$

Where ${GFR}_{birth}$ is the GFR at birth,${TVGFR}_{birth}$ is the GFR at birth for a typical individual with a birthweight of 1,750 g, ${GFR}_{max}$ is the maximum GFR,${PNA}_{50}$ is the PNA at which 50% of ${GFR}_{max}$ is reached, and γ is the Hill coefficient.

To connect Scr to GFR (Eq. 2), published eGFR equations (*i.e.,* the Schwartz bedside equation and adjusted Schwartz equations [26–29, 31, 32]) that were based on mechanistic principles eGFR = synthesis rate/Scr, and were built on data from children, were transformed to generate the synthesis rate (Table S1). The Schwartz bedside equation [28] was used when developing the base model. To select the most appropriate eGFR function, after all covariates were selected, the other synthesis rate equations were tested again and compared to the Schwartz bedside equation based on the numerical (objective function values (OFV)) and visual (goodness of fit (GOF) plot) assessments. A difference in the OFV between 2 hierarchical models was assumed to follow a *χ*^2^ (chi-square) distribution, and for 1 degree of freedom, a decrease in OFV of 10.83, corresponding to a significance level (α) of 0.001, was taken to be statistically significant.

#### Stochastic Model

The stochastic model included interindividual variability (IIV) and residual variability (RUV). The IIV on ${GFR}_{birth}$, ${GFR}_{max}, {PNA}_{50},$ γ were implemented assuming a log-normal distribution [52]. For RUV, inulin CL observations were log-transformed, and a constant additive residual error was assumed. A combined proportional and additive residual error model was applied for the Scr observations.

#### Covariate Analysis

A covariate analysis was conducted on the parameters ${PNA}_{50}$ and ${GFR}_{max}$ in Eq. 4. For continuous covariates (birthweight, current weight, GA, PNA, PMA), linear, power and exponential functions were tested to describe the relationship between covariates and parameters. For categorical covariate (sex), additive shift models were tested. Covariates were evaluated based on OFV by a stepwise forward inclusion (ΔOFV > 6.64, P < 0.01) and backward deletion (ΔOFV < 10.83, P > 0.001) process. Besides this, GOF plots split for covariate quartiles were assessed to check whether the covariate model results in an unbiased description of the different quartiles.

##### Covariate Analysis for ${{\varvec{P}}{\varvec{N}}{\varvec{A}}}_{50}$

Birthweight, GA and PMA, as criteria for prematurity, were first tested as covariates for ${PNA}_{50}$. A previous study has shown that preterm neonates mature more slowly than term neonates [12]. In case any of these parameters was a significant covariate on *PNA*_*50*_, it could be assumed that the rate of postnatal maturation differed between neonates with different degrees of prematurity. On top of this, the influence of sex on ${PNA}_{50}$ was examined.

##### Covariate Analysis for ${{\varvec{G}}{\varvec{F}}{\varvec{R}}}_{{\varvec{m}}{\varvec{a}}{\varvec{x}}}$

The influence of current weight, BSA and FFM on ${GFR}_{max}$ was tested, as GFR is known to be influenced by body size [57]. Additionally, birthweight and GA were tested as predictors of ${GFR}_{max}$ to observe if prematurity has a lasting effect on GFR. If these covariates showed an effect on ${GFR}_{max}$, the maximum value that can be reached by preterm neonates would be different to their term peers after size differences had been accounted.

#### Model Evaluation

Besides above mentioned criteria, model evaluation was conducted by plotting conditional weighted residuals (CWRES) *versus* PNA, split by GA groups. A normalized prediction distribution error (NPDEs) analysis was performed based on 1,000 simulations to assess the predictive ability of the final model. Each observation was compared to the range of simulated values using the NPDE package in R [58].

#### Model Simulation

The final GFR maturation model was used to simulate GFR for four typical individuals from birth to 18 years old, born with a GA of 26, 30, 35, and 40 weeks, corresponding to birthweights of 850, 1,500, 2,500, and 3,500 g, respectively. The simulated current weight and height for those individuals were calculated using the mean predictions for boys and girls from the developed growth curve (described in the Supplementary materials).

### Application of GFR Maturation Model to Describe the Maturation of Drug Clearance

To evaluate whether the developed GFR maturation model can be used for describing the maturation of drug CL across a wide population, we used the data of De Cock *et al. *[59] who developed a joint covariate model to describe the maturation of CL of the three renally cleared drugs gentamicin, vancomycin and tobramycin. The three datasets for the three different drugs in this publication [60–63] were used for the development of respective popPK models, in which the developed GFR function was used as a covariate function on CL. In the three drug datasets, GA or birthweight were not recorded for 44(6%), 98(16%), and 91(21%) individuals, respectively. When these values were missing in individuals below 1 year of PNA, the patients were removed from the dataset, as imputation was expected to potentially result in bias. For 15(2%), 71(12%), 55(14%) patients aged above 1 year with missing GA in gentamycin, tobramycin and vancomycin dataset, respectively, GA was assumed to be 40 weeks. The demographic information for the three datasets is provided in Table S2.

In the development of the popPK model for the three drugs, the respective base model and stochastic model (Table S3) from de Cock *et al. *[59] were applied. In the covariate analysis, each drug's population CL was fixed separately to an estimated fraction of the fixed GFR equation.

5 $$CL=F\times GFR$$

Where F, the estimated fraction and volume of distribution were considered to contain drug-specific information and were therefore estimated for each drug separately. The underlying assumption for allowing for CL to be a fraction of the estimated GFR and thus higher or lower than GFR was that for the different drugs, reabsorption and secretion and the free fraction of the drug could play a role in determining CL.

Evaluation of the predictive properties of the models using the GFR covariate model was assessed by GOF plots categorized by different GA groups.

## Results

### Dataset

The final dataset yields a total of 431 inulin CL values from 383 children, with a median GA of 36 (range: 25–43) weeks, PNA 4 (0–6,570) days, and current weight of 2.1 (0.5–83.3) kg. There were 2,181 Scr concentrations from 394 children with median GA of 29 (23–44) weeks, PNA 185 (0–6,459) days, and current weight of 5.8 (0.4–74.4) kg.

**Fig. 1:**
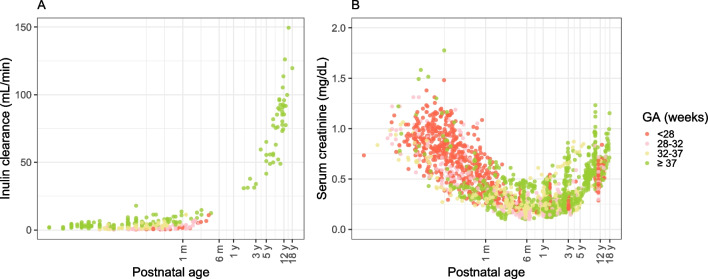
Observed data of inulin CL (**A**) and serum creatinine concentrations (**B**) *versus* postnatal age, colored by gestational age (GA). The x-axis is on a log-scale.

The demographic information of the included patients is shown in Table 1. Inulin CL data was primarily available from infants below 107 days of PNA, with no available data for preterm-born individuals beyond that age. In contrast, Scr concentrations were available across the entire range of both GA and PNA. The raw plot of inulin clearance and Scr concentrations are shown in Fig. 1. Inulin CL increases with PNA and there is a clear difference in inulin CL between different GA groups in the first 3 months of life. Scr concentrations decrease until around 1 year of age, after which Scr increases.

**Table 1:** Demographic Information of the Patients Included in the Inulin Clearance Dataset and Serum Creatinine Concentration Dataset

	Inulin clearance	Serum creatinine concentration			
		Dataset 1	Dataset 2	Dataset 3 [48]	Dataset 4 [17]
Number of IDs, *n*	383	71	98	125	100
Number of samples, *n*	431	1,391	565	125	100
Sample(s) per ID (*n*)	1 (1–4)	11 (1–137)	5 (1–20)	1	1
Birthweight (g)	2,300 (580–4,950)	2,168 (525–4,270)	943 (465–2,185)	2,470 (430–5,000)	965 (580–4,680)
Current Weight (kg)	2.1 (0.50–83.3)	8.2 (0.50–74.4)	1.0 (0.4 – 4.8)	36.4 (21.4–71.4)	34.7 (19.0–68.0)
BSA (m^2^)	0.17 (0.07–1.99)	0.41 (0.07–1.93)	0.10 (0.06–0.27)	1.21 (0.85–1.83)	1.17 (0.81–1.75)
Length (cm)	47 (35–170)	71 (24–181)	35 (21–54)	147 (124–182)	144 (123–163)
Gestational age (week)	36.0 (25.0–43.0)	35.4 (24.4–42.0)	26.9 (24.1 – 31.9)	37 (24.0–42.0)	29.5 (23.0–44.0)
Postnatal age (days)	4 (0–6,570)	416 (0–6,459)	8 (1 – 229)	4,170 (3,379–5,425)	4,015 and 3,906
GA ≤ 28 weeks	10 (1–107)	30 (0–1,505)	8 (1–229)	4,174 (3,484–5,275)	4,015
GA 28–32 weeks	5 (1–66)	205 (0–1,901)	9 (1–118)	4,350 (3,504–5,373)	4,015
GA 32–37 weeks	3 (1–35)	295 (0–4,827)	-	4, 819 (4,425–5,212)	4,015
GA 37–42 weeks	3 (0–6,570)	617 (1–6,459)	-	4,052 (3,379–5,425)	3,906
Postmenstrual age (weeks)	36.2 (25.4–978.6)	96.1 (24.6–960.7)	28.0 (24.4–60.6)	625.7 (521.7–813)	598.9 (594.9–605.6)
Sex, *n*					
Boy	154	35	52	66	40
Girl	229	36	46	59	60
Duration of follow-up (days)	1 (1–33)	604 (1–2,063)	19 (1–226)	1 (1–1)	1 (1–1)

All values are indicated as median (range) unless stated otherwise. *BSA* body surface area; *GA* gestational age

### Model Development

As mentioned in the Methods, GFR maturation until birth (${GFR}_{birth}$) was described by a linear function with birthweight, and postnatal maturation was described by a sigmoidal Emax function with key parameters ${GFR}_{max}$,${PNA}_{50}$ and $\gamma$ (Eq. 4). IIV could be estimated for ${GFR}_{birth}$, ${GFR}_{max}$,${PNA}_{50}$ and $\gamma$. In the covariate analysis, GA was the best covariate to describe the negative effect of prematurity on ${PNA}_{50}$, implying that the higher the GA, the faster the GFR matures. No additional covariates were found to be statistically significant on ${PNA}_{50}$. For ${GFR}_{max}$, BSA in a power equation with an exponent of 1.06 was found to be the best parameter. However, compared to the current weight with an estimated exponent of 0.738, it only reduced OFV by 9.2 points. Considering the minor difference and the fact that current weight is more commonly used, the model with current weight was selected as the final model. GA and sex were not found to be statistically significant covariates on ${GFR}_{max}$.

**Fig. 2:**
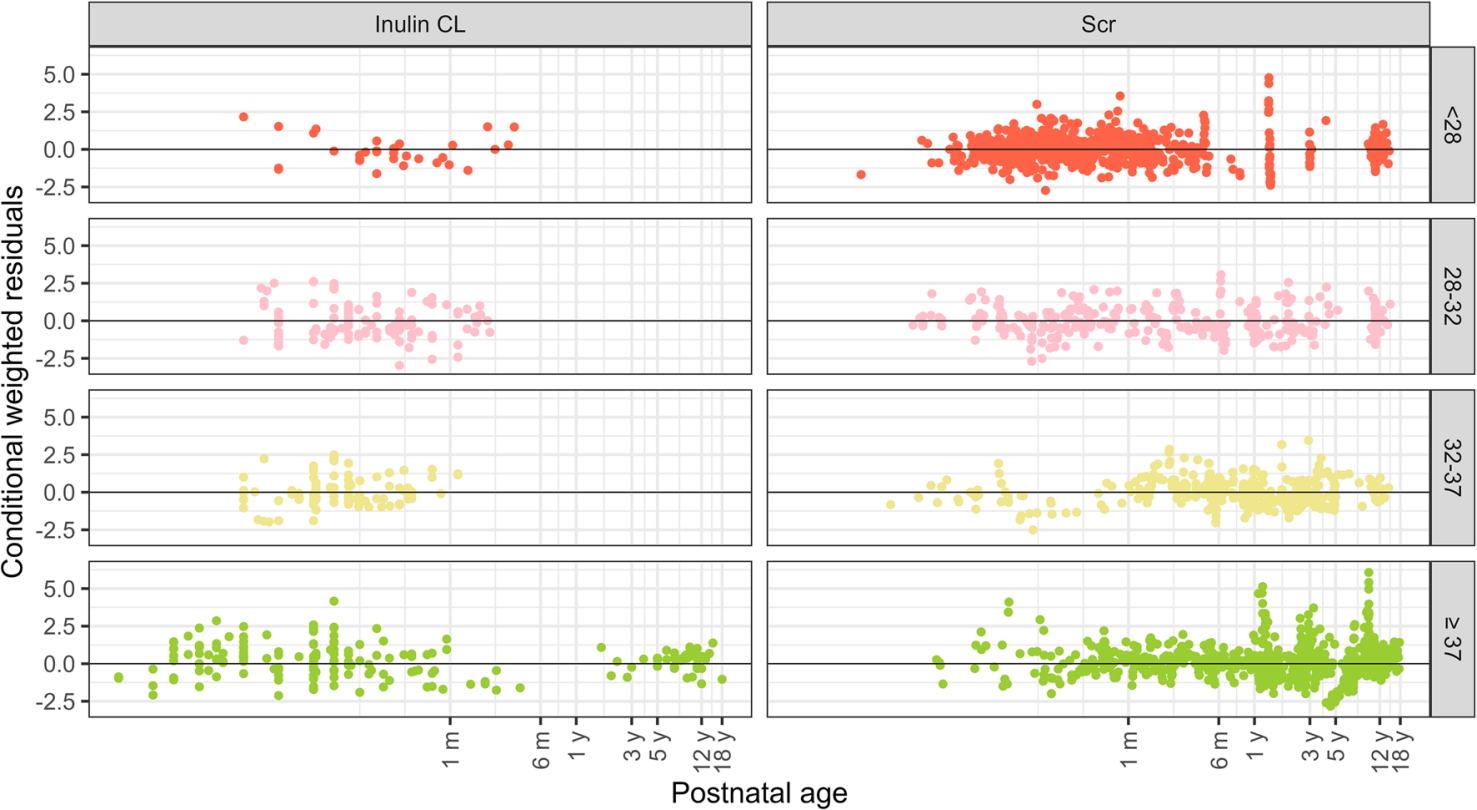
Conditional weighted residuals of the final model *versus* postnatal age (PNA) for the different GA groups*, i.e.,* < 28, 28–32, 32–37 and ≥ 37 weeks, split by inulin clearance (CL, left panel) and serum creatinine (Scr, right panel) concentrations. The x-axis is on a log-scale.

The final model and parameter estimates are shown in Table 2. The split goodness of fit plots (Fig. 2) did not show obvious model misspecification across PNA ranges in different GA strata for either inulin CL or Scr concentrations. No structural trends were observed in NPDEs when plotted against PNA, birthweight, GA, current weight, and predicted values (Supplementary Figure S1).

**Table 2:** Parameter Estimates of the Final GFR Maturation Model

Parameter	Parameter estimate (RSE %)	IIV as CV% (RSE%) [shrinkage %]
Fixed effects		
$$GFR={TVGFR}_{birth}\times \frac{Bwb}{1750}+ \frac{\left(TV{GFR}_{max}\times {\left(\frac{CW}{1750}\right)}^{{\text{fCW}}}-{TVGFR}_{birth}\times \frac{Bwb}{1750}\right)\times {PNA}^{\gamma }}{{\left(\frac{GA}{34}^{GA{PNA}_{50}}\times TV{PNA}_{50}\right)}^{\gamma }+ {PNA}^{\gamma }}$$		
${TVGFR}_{birth}$(mL/min)	1.26 (4)	31.1 (16) [57]
$TV{GFR}_{max}$(mL/min)	8.98 (12)	17(10) [47]
${\text{f}}CW$	0.738 (5)	-
$TV{PNA}_{50}$(days)	34 (31)	55.9 (43) [57]
$GA{PNA}_{50}$	-3.61 (18)	-
$\gamma$	1.03 (7)	35.5 (20) [55]
Residual errors		
Parameters	Parameter estimate (RSE %)	Shrinkage %
σ2 Inulin CL-log additive	0.033 (55)	43
σ2 Scr-additive	0.00187 (36)	10
σ2 Scr-proportional	0.0198 (40)	10

*Bwb* birthweight (gram); *CL* clearance; *CV* coefficient of variation; *CW* current weight (gram); *GA* gestational age (weeks); *GFR* glomerular filtration rate; *IIV* inter-individual variability; *PNA* postnatal age (day); *RSE* relative standard error

In addition to the Schwartz bedside equation [28], other published eGFR equations were tested to connect Scr with GFR. When using the Pierce equation [32] to describe the synthesis rate, OFV was the lowest among the other equations, which had a 43-point reduction when compared to the Schwartz bedside equation [28]. At the same time, it corrected an underprediction of Scr in children between 12–18 years of age (Figures not shown), and therefore, the Pierce equation [32] was kept in the final model as the connection between Scr and GFR (Eq.2).

**Fig. 3:**
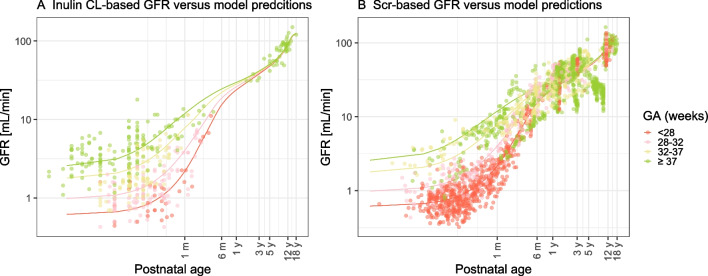
Final GFR model based predictions (solid lines) *versus* postnatal age (PNA) for four typical individuals with GA of 26, 30, 35, 40 weeks with corresponding birthweight of 850, 1500, 2500 and 3500 g, against observed inulin clearance (CL) values (dots) (**A**) and against Serum creatinine (Scr) observation based estimated glomerular filtration rate (eGFR) using Pierce equation [32] (dots) (**B**) with lines and dots colored by gestational age (GA). The x-axis is on a log-scale.

**Fig. 4:**
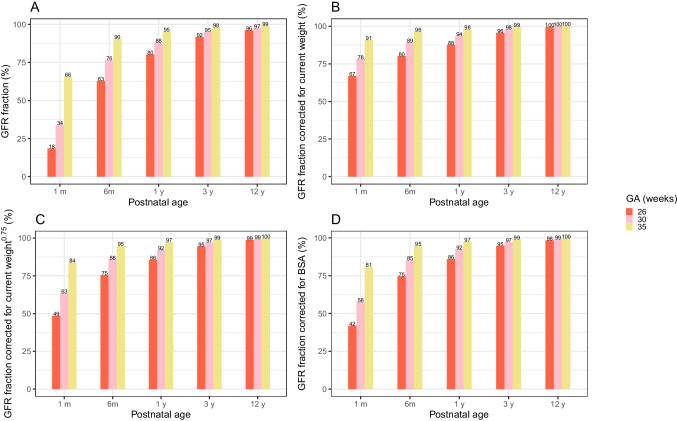
Fraction of population predicted value for glomerular filtration rate (GFR) of typical individuals each born prematurely at a gestational age (GA) of 26, 30, or 35 weeks, respectively, compared to a typical individual born term with a GA of 40 weeks at the same postnatal age. The GFR is expressed in absolute value in mL/min (**A**), mL/min/kg (**B**), mL/min/kg^0.75^ (**C**), or mL/min/BSAm^2^ (**D**).

Figure 3 illustrates the predictions based on the final GFR maturation model (solid lines) *versus* PNA against observed inulin CL values (dots) (**A**) and *against* Scr observation-based eGFR using Pierce equation [32] (dots) (**B**) in typical preterm-born and term-born individuals. Below 1 year of age, the largest differences in the absolute GFR values between different GA groups are seen. From this figure, it becomes clear that GFR in individuals born preterm catches up at around 3 years of age. Figure 4 illustrates the fraction of population predicted value for GFR in mL/min (**A**) or mL/min/kg (**B**) or mL/min/kg^0.75^ (**C**) or mL/min/BSA m^2^ (**D**) of each typical preterm-born individual compared to their 40-week term-born peers. Taking an individual born at a GA of 26 weeks as an example, its absolute GFR (**A**) reaches 18%, 63%, 80%, 92%, and 96% of the GFR of a term-born (40 weeks) peer at 1 month, 6 months, 1 year, 3 years, and 12 years, respectively. After correcting for current weight (**B**), these fractions are 67%, 80%, 88%, 96%, and 100%, respectively. After correcting for current weight based on allometric scaling(**C**), these fractions are 49%, 75%, 86%, 95%, and 99%, respectively. After correcting for BSA (**D**), these fraction are 42%, 75%, 86% 95% and 98%, similar to the fraction after correcting the current weight differences in an allometric scaling.

### Application of GFR Maturation Model to Describe the Maturation of Drug Clearance

**Fig. 5:**
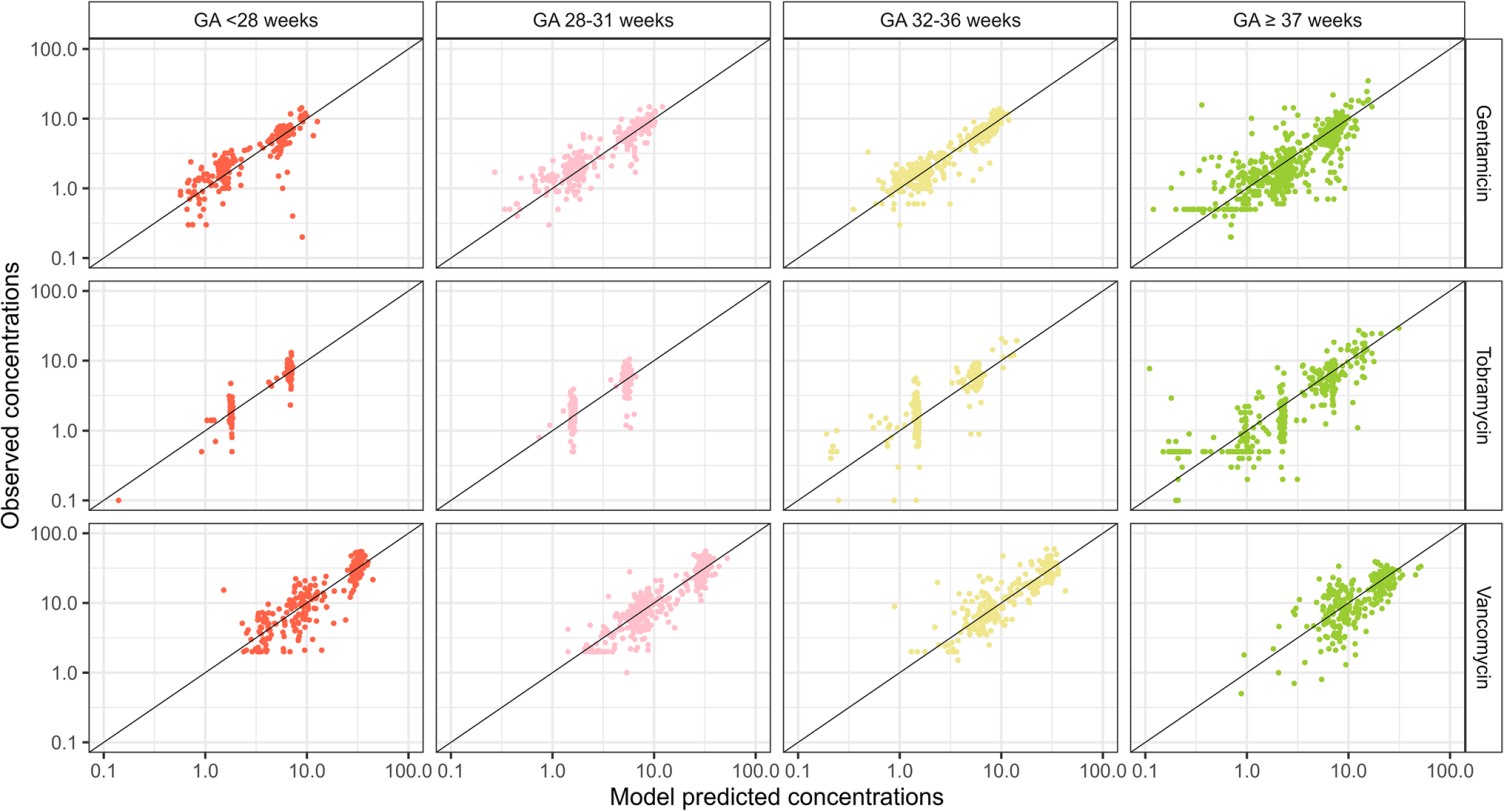
Observed *versus* population predicted concentrations of the pharmacokinetic model with GFR maturation function predicted-clearance for gentamicin, tobramycin or vancomycin, split by four gestational (GA) groups.

The model structure and parameter estimates for the PK models implementing the GFR function on CL of gentamicin, tobramycin and vancomycin are listed in Table S3. The estimated fractions of the CL of gentamicin, tobramycin, and vancomycin to GFR were 0.62, 0.74, and 0.67, respectively. The estimates of volume and other fixed effect parameters were all within 10% of those of the models published by de Cock *et al. *[59]. Compared to the models reported by de Cock *et al. *[59], the OFV changed by -219, + 22 and -151 points for gentamicin, tobramycin and vancomycin, respectively. The observed concentrations *versus* population-predicted concentrations of those three drugs, split by GA groups, are shown in Fig. 5, and the CWRES *versus* PNA plots split for GA groups are shown in Figure S2-S4. The concentrations of all three drugs are generally well predicted across ages and, in line with the lower or similar OFVs for the three models, the description seems superior compared to the published models by de Cock *et al. *[59].

## Discussion

Using a novel approach of leveraging information from inulin clearance and Scr concentrations in one analysis, we built a GFR maturation equation for preterm and term-born children from birth to 18 years of age. Adding Scr data was crucial to provide information where inulin data were unavailable, *i.e.,* for prematurely born individuals older than 3 months. The results show that the difference in GFR between preterm and term-born children is very large at birth and minimizes at around 3 years of age, with the fastest catch-up of GFR in preterms in the first year of life. The successful application of this GFR maturation equation to the CL of popPK models of renally cleared drugs shows that the concentration predictions using the GFR maturation equation were similar or even superior to that of a previously developed model by De Cock *et al. *[59]. This indicates that the GFR maturation equation can be used as a covariate function in popPK analyses in sparse neonatal and pediatric data analyses and be a novel tool to propose doses for renally excreted drugs for preterm and term-born infants. Still, further application of this GFR maturation function to other renally cleared drugs, whether primarily cleared through glomerular filtration or also cleared by active secretion or reabsorption, would be relevant to test the general applicability of this model. However, this analysis is beyond the scope of this paper.

Little attention has been given to the dosing requirements of preterm-born infants beyond the first month of life. This study represents a pioneering effort to address this gap in knowledge, marking the initial step toward understanding and optimizing medication dosing for this specific population. The GFR maturation equation suggests that, when dosing based on current weight (mg/kg), preterm neonates with a GA of 26 weeks at 1 month of age may still require a dosage reduction to 67% of that administered to term neonates, while at 6 months of age, the dosage may need to be reduced to 80% of the term neonatal dose (see Fig. 4B). The fraction based on current weight in an allometric scaling in Fig. 4C is similar to the values we see in Fig. 4D when corrected by BSA. This similarity is the result of similar contribution of BSA in a linear scaling and current weight in an allometric scaling as a size factor to the GFR maturation. We also compared the differences in scaling methods when extrapolating GFR clearance from 18 years old to birth, by comparing current weight allometric scaling and linear scaling to the GFR maturation function (Figure S5). It can be observed that allometric scaling fitted for older children while linear scaling resulted in closer prediction of GFR in neonates. This is in line with the conclusions from the study of Cristea *et al. *[64] and Krekels *et al.* [65]*.*

Our study found that an individual born at GA 26 weeks reaches around 42%, 75%, 86%, 95%, and 98% of the GFR in mL/min/m^2^ as their term-born peers at postnatal age 1 month, 6 months, 1 year, 3 years, and 12 years, respectively (Fig. 4D). Similarly, at around 9 months and 24 months of corrected age, Vanpee *et al*. and Gheissari *et al. *[13, 14] found a significant difference in BSA-corrected GFR between low-birthweight infants and term-born controls. Studies that focused on older children, however, yielded ambiguous results. Most of the studies claimed no difference in BSA-corrected (e)GFR at 5 [16], 5–10 [15], 8 [13], 9–12 [19], and 20 years [18]. Some studies detected a statistically significant but small difference among a group of term controls, children born preterm and appropriate for GA, and children born preterm and small for GA at 11 years (110.0, 106.8 and 99.0 mL/min/1.73m^2^, respectively) [20] and between preterm and term-born children at 14 years (126.2 *versus* 134.3 mL/min/1.73m^2^) [21].

A similar GFR in preterm and term-born children in late childhood does not exclude the possibility that the renal function of preterm-born individuals more rapidly declines with age during adulthood. Preterm-born individuals are known to be born with lower nephron numbers [8, 9], meaning that they achieve the same GFR function as term-born individuals by greater utilization of the limited number of functional units of the kidney, implying a potential increase of single nephron filtration rates and hyperfiltration [19, 66]. Glomerular hypertrophy could potentially explain the lack of significant differences in kidney volumes between preterm and term individuals in childhood after adjustment for BSA, sex, and age [16]. All of this could eventually make them vulnerable to a second hit, as researchers have established a link between prematurity/low birthweight and an increased risk of hypertension and chronic kidney disease later in life [67, 68].

The model developed in this study is an extension of the previously published GFR maturation function [12] that separately describes the prenatal and postnatal GFR maturation in neonates and infants under 90 days of age based on inulin data. Recently, another model on the effects of birth has been published by O’Hanlon *et al. *[55]. The authors considered the steep increase of GFR caused by birth by introducing an asymptotic exponential PNA maturation equation as an addition to a PMA and FFM-based sigmoidal maturation function. Their equation related to PNA asymptotically approaches 1 at 35 days of life (5 times maturation half-life), after which the authors assumed that the disparity between preterm and term neonates is solely attributed to PMA and FFM. In other words, the authors assumed that for two children with the same PMA and FFM but differing PNA values due to preterm birth, the one who is born preterm will always have higher GFR because the PMA-based GFR component is identical, but the PNA-based GFR component will be greater in the preterm-born child, because of the higher PNA of this child. On the other hand, in the current study, we found that the postnatal maturation rate depends on GA (Fig. 3). One strength of our study is that we included GFR marker data of preterm neonates from birth to 18 years old, which provides more solid information for describing GFR maturation later in childhood in this vulnerable population. In addition, the good predictive performance of our model in renally cleared drugs underlines the validity of our assumption.

The GFR maturation model developed in this work features four IIV parameters, namely on ${GFR}_{birth}$, ${GFR}_{max}$, ${PNA}_{50}$ and$\gamma$. To be able to quantify these IIVs in maturational trajectories, we had longitudinal data of several Scr values over time within subjects. In our model, the impact of IIV on the maturation parameters on GFR changes as a function of age and is strongest for the youngest children, which is to be expected as PK variability is usually higher in neonates compared to adults [69, 70]. For example, in a developmental PK model of oxycodone from neonates to adults [71], the authors quantified that unexplained IIV in oxycodone clearance after IV administration is about 60% at the time of birth and approaches about 30% at maturity. In typical popPK analyses, only a single IIV is used to describe CL variability for each patient, and the IIV is assumed to be constant with covariates such as age. We refrain from making strong statements on whether popPK models should routinely investigate whether the extent of IIV is dependent on covariates. Nonetheless, we do point out that both our analysis and empirical observations suggest that the extent of IIV is not necessarily constant across the population, and most popPK models rarely capture this. The clinical implication is that when giving renally cleared drugs, even when maturation factors are accounted for, dosing individualization by measuring the kidney function may be of relevance, particularly in the youngest individuals.

Our study showed that Scr concentrations decrease until around 1 year of age, after which Scr concentration increases (Fig. 1). This pattern aligns with observations from previous studies [50, 72, 73], demonstrating the age-related, dynamic interplay between the synthesis rate of creatinine and GFR; Specifically, Scr concentration decreases in the first year of life, when the rate of increase in GFR with age outpaces that of the creatinine synthesis rate. Conversely, after one year, the rate of increase in GFR slows relative to the synthesis rate, leading to an increase in Scr concentrations.

Our methodology uses a Scr-based eGFR function to relate Scr concentrations to GFR. The equation by Pierce *et al. *[32] was selected as the best descriptor for this relationship, as it optimally fitted for the Scr concentrations and inulin CL data. Compared to the Schwartz bedside equation [28], which uses a single constant to describe the relationship between GFR and a child’s height-to-serum creatinine ratio, the eGFR function developed by Pierce *et al. *[32] incorporates age and sex-dependent constants into this relationship. Consequently, it predicts higher synthesis rates for children aged between 12–18 years compared to Schwartz's bedside equation and could correct an underprediction of Scr in this age group. Smeets *et al. *[74] compared the eGFR calculated from the Pierce equation and Schwartz bedside equation to iohexol-based measurements and also showed that the Pierce equation was superior in term-born neonates and children.

The underlying assumption for using the creatinine-based eGFR equations is that the system is in (quasi) steady-state, wherein the formation rate of creatinine is equivalent to its elimination rate or when the half-life of creatinine is significantly shorter than the observation time gap. However, this assumption may pose a challenge for neonates during the first 4 days of life, as their renal CL is extremely low, potentially resulting in a higher production rate of creatinine than its elimination rate. This is, for example, reflected in the abrupt rise of its concentration at around 4 days, as published by others [56, 75]. However, this does not seem to undermine the robustness of the GFR maturation model presented in this paper, as the model during this period is well-informed by the inulin CL data (Fig. 1), and the GOF plots of both Scr and inulin CL generally fit well (Fig. 2). Another situation when this assumption fails is when GFR falls abruptly, such as in AKI. Therefore, the subjects undergoing AKI were deleted from the analyzed dataset, irrespective of the specific characteristics (disease conditions like asphyxia, or sepsis, postoperative setting, or co-medications). Other groups that could violate this assumption would be children with chronic kidney disease or with muscle disease, however we anticipate those numbers to be low and of less relevance. We did not exclude Scr concentrations from previously defined AKI subjects, as parameter estimates of models with and without those concentrations changed very little (less than 10%).

## Conclusions

Using a novel approach analyzing inulin CL values and Scr concentrations across the pediatric age range, a GFR maturation model for term and preterm-born individuals was successfully developed throughout childhood. The predictions based on the model showed significant GFR differences below 1 year, with preterm-born individuals reaching at least 80% of the GFR of their term-born peers at 1 year of age. The difference in GFR between preterm and term-born individuals diminishes at 3 years of age. This GFR maturation model can be used to inform the maturation of the clearance of renally cleared drugs and facilitate the dosing of these drugs for preterm-born individuals.

### Supplementary Information

Below is the link to the electronic supplementary material.

Supplementary file1 (DOCX 930 KB)
