# Supplementary material for: When will the Glomerular Filtration Rate in Former Preterm Neonates Catch up with Their Term Peers?
Source: Pharm Res. 2024 Mar 12;41(4):637–49. doi: 10.1007/s11095-024-03677-3 (PMC11024008; doi:10.1007/s11095-024-03677-3)
Supplement: Supplementary file 1 — Supplementary file1 (DOCX 930 KB) [file 11095_2024_3677_MOESM1_ESM.docx]

**When will the Glomerular Filtration Rate in former preterm neonates catch up with their term peers?**

**Supplementary materials**

Yunjiao Wu (1), Karel Allegaert (2,3), Robert B. Flint (2,4), Sebastiaan C Goulooze (5), Pyry A J Välitalo (6,7), Matthijs de Hoog (8), Hussain Mulla (9), Catherine MT Sherwin (10), Sinno H.P. Simons (4), Elke H.J. Krekels (1, 11), Catherijne A.J. Knibbe (1,4,12), Swantje Völler (1,4)*

1. *Division of* *Systems Pharmacology and Pharmacy, Leiden Academic Centre for Drug Research, Leiden University, Leiden, The Netherlands.*

*2. Department of Hospital Pharmacy, Erasmus University Medical Center, Rotterdam, The Netherlands*

*3. KU Leuven, Department of Development and Regeneration, and Department of Pharmaceutical and Pharmacological Sciences, Leuven, Belgium*

*4. Department of Pediatrics, Division of Neonatology, Erasmus MC Sophia Children's Hospital, Rotterdam, The Netherlands*

*5. Leiden Experts on Advanced Pharmacokinetics and Pharmacodynamics (LAP&P), Leiden, The Netherlands.*

*6. School of Pharmacy, University of Eastern Finland, Yliopistonranta 1 C, 70210 Kuopio, Finland*

*7. Finnish Medicines Agency, Hallituskatu 12-14, 70100 Kuopio, Finland*

*8. Department of Neonatal and Pediatric Intensive Care, Division of Pediatric Intensive Care, Erasmus MC Sophia Children's Hospital, Rotterdam, The Netherlands*

*9, Department of Pharmacy, University Hospitals of Leicester, Glenfield Hospital, Leicester, LE39QP, England*

*10, Department of Pediatrics, Wright State University Boonshoft School of Medicine/Dayton Children’s Hospital, One Children's Plaza, Dayton, Ohio, USA*

*11, Certara Inc, Princeton, NJ, USA*

*12. Department of Clinical Pharmacy, St Antonius Hospital, Nieuwegein, The Netherlands.*

***Corresponding author**

Swantje Völler, PhD

Division of Systems Pharmacology and Pharmacy, Leiden Academic Center for Drug Research

Leiden University

2333CC Leiden

The Netherlands

Telephone: +31 71 527 6331

E-mail: [s.voller@lacdr.leidenuniv.nl](mailto:s.voller@lacdr.leidenuniv.nl)

**Generation of bodyweight and height growth curves**

We created current weight and height curves in relation to postmenstrual age (PMA) for various gestational age (GA) groups and both sexes, spanning from birth to 18 years. These curves were constructed by connecting the median weight data from growth charts that cover different ranges of age groups. This includes growth chart for preterm[1] and term neonates[2] with the description of bodyweight loss during first weeks of life, and growth chart from 50 weeks of PMA to 19 years old provided by *'2014 WHO Growth Charts for Canada with revisions based on re-analysis by the Canadian Pediatric Endocrine Group’*[3]. Our assumption was that individuals born at different GA would eventually reach the same bodyweight at 50 weeks of PMA. B-splines, using bs function in the splines package in R, were used to fit the median data points and smooth the gaps between different growth charts. For height *versus* PMA curves, neonates with same PMA were assumed to have the same height. The median height *versus* PMA curves from revised Fenton [4] and WHO[5] charts were combined and fitted using a B-spline.

**Imputation of missing bodyweight and height**

For the missing bodyweight observation in serum creatinine concentration that could not be interpolated, if the time gap between the nearest weight observation and the missing observation was shorter than 10 days, the nearest observation was extrapolated. Otherwise, the missing values were predicted based on the trend of standard weight growth chart, after adjusting by the median ratio between available weight and weight from the standard growth curve.

| $Ratio,j=Median(\frac{{CW}_{obs,i,j}}{{CW}_{growth chart, i, j}})$ | Eq.1 |
| --- | --- |
| ${CW}_{missing,i,j}={CW}_{growth charti, j}*Ratio,j$ | Eq.2 |

Where $Ratio,j$ is the calculated ratio for *jth* subject, ${CW}_{obs,i,j}$ is the *ith* observed current weight for the *jth* subject, the ${CW}_{growth chart, i, j}$is the predicted *ith* current weight for *jth* subject using growth chart, ${CW}_{missing,i,j}$ is the *ith* missing current weight for *jth* subject.

The imputation of missing height is similar to that of current weight, only the maximum time for extrapolation was extended to 60 days.

**Table S1 Published serum creatinine synthesis rate functions**

| **Reference** | **Equations** |
| --- | --- |
| Schwartz[6, 7] | k * Height *(BSA/1.73)  Age ≤ 1year k = 0.33 for preterm k= 0.45 for term  Age 1-12 years k=0.55  Age 13-21 years k=0.55 for female and k=0.7 for male |
| Schwartz Bedside[8] | 0.413 * Height *(BSA/1.73) |
| Schwartz Adapted by Holness [9] | 0.330* Height *(BSA/1.73) |
| Schwartz-Lyon[10] | Girls and boys <13 years: 0.368* Height *(BSA/1.73)  Boys ≥13 years: 0.413×height*(BSA/1.73) |
| Pierce 2021[11] | k * Height / 88.4*(BSA/1.73)  For males, k is calculated as:  For 1 to <12 years old: 39.0*1.008^ (age-12)  For 12 to <18 years old: 39.0*1.045^ (age-12)  For 18 to 25 years old: 50.8  For females, k is calculated as:  For 1 to <12 years old: 36.1*1.008^ (age-12)  For 12 to <18 years old: 36.1*1.023^ (age-12)  For 18 to 25 years old: 41.4 |

The unit for synthesis rate is mg/min*100, the unit of height is in cm, the unit of BSA is m^2^ (BSA = body surface area), age in years.

**Table S2 Overview of the patient characteristics of the final drug model**

|  | **Gentamycin[12, 13]** | **Tobramycin[14]** | **Vancomycin[15]** |
| --- | --- | --- | --- |
| **Number of IDs, n** | 688 | 587 | 393 |
| **Number of samples, n** | 1,611 | 1,211 | 1,066 |
| **Sample per ID (n)** | 2 (1-10) | 2 (1-8) | 2 (1-22) |
| **Birthweight (g)** | 2,380 (440-5,240) | 1,750 (485-5,245) | 1,435 (385-4,590) |
| **Current Weight (kg)** | 2,380.0 (440.0-80,000.0) | 1,800.0 (485.0-85,000.0) | 1,500.0 (415.0-85,000.0) |
| **Gestational age (week)** | 34.5 (23.0-42.0) | 32.4 (23.7-42.8) | 31.0 (23.0-43.0) |
| **Postnatal age at start of study (days)** | 2 (1-5,483) | 3 (2-6,548) | 15 (1-6,121) |
| GA ≤ 28 weeks | 2 (1-64) | 3 (3-4,867) | 11 (2-336) |
| GA 28–32 weeks | 2 (1-13) | 3 (3-168) | 12 (2-810) |
| GA 32–37 weeks | 2 (1-58) | 3 (3-4,739) | 12 (1-2,345) |
| GA 37–42 weeks | 3 (1-5,483) | 48 (2-6,548) | 1,208 (4-6,121) |

All values are indicated as median (range) unless stated otherwise. GA, gestational age.

**Table S3 Parameter estimates of the final drug models**

| **Parameter** | **Parameter estimate**  **(RSE %)** | **IIV as CV% (RSE%) [shrinkage %]** |
| --- | --- | --- |
|  |  |  |
| **Fixed effects** | | |
| $\boldsymbol{CLdrug}\boldsymbol{=f\times}\boldsymbol{GFR}$ | | |
| f, gentamicin | 0.617 (2) | 34.8 (7) [17] |
| f, tobramycin | 0.737 (2) | 39.6 (8) [14] |
| f, vancomycin | 0.668 (2) | 35.8 (7) [15] |
| $\boldsymbol{V}\boldsymbol{1}\boldsymbol{genta=}\boldsymbol{V}_{\boldsymbol{4}\boldsymbol{kg}}\boldsymbol{\times}{\boldsymbol{(CW/4}\boldsymbol{kg)}}^{\boldsymbol{k}}$ | |  |
| $V_{4kg}$, gentamicin | 1.56 (3) | - |
| k | 0.823 (4) | - |
| $\boldsymbol{V}\boldsymbol{1}\boldsymbol{tobra=}\boldsymbol{V}_{\boldsymbol{4}\boldsymbol{kg}}\boldsymbol{\times}{\boldsymbol{(CW/4}\boldsymbol{kg)}}^{\boldsymbol{k}}$ | | |
| $V_{4kg}$, tobramycin | 1.87(2) | - |
| k | 0.749(2) | - |
| $\boldsymbol{V}\boldsymbol{1}\boldsymbol{vanco=}\boldsymbol{V}_{\boldsymbol{4}\boldsymbol{kg}}\boldsymbol{\times}{\boldsymbol{(CW/4}\boldsymbol{kg)}}^{\boldsymbol{k}}$ | | |
| $V_{4kg}$, vancomycin | 2.3 (3) | - |
| k | 1.05 (2) | - |
| Qgenta=CLgenta  Qtobra=CLtobra  Qvanco=CLvanco  V2genta=V1genta  V2tobra=V1tobra  V2vanco=V1vanco |  |  |
| **Residual variability** | | |
| **Parameter** | **Parameter estimate (RSE %)** | **Shrinkage %** |
| σ^2^gentamicin | 0.0899 (9) | [13] |
| σ^2^tobraycin, proportional  σ^2^tobraycin, additive | 0.0571 (9)  0.0442 (22) | [18]  [18] |
| σ^2^vancomycin | 0.103 (8) | [11] |

CL, clearance; CV, coefﬁcient of variation; CW, current weight; f, fraction of drug clearance of glomerular filtration rate; GA, gestational age; GFR, glomerular filtration rate; IIV, inter-individual variability; Q, intercompartmental clearance; RSE, relative standard error. V1 volume of distribution of the central compartment, V2 volume of distribution of the peripheral compartment. k, the exponent of current weight on V1. σ^2^ , variance of residual error.


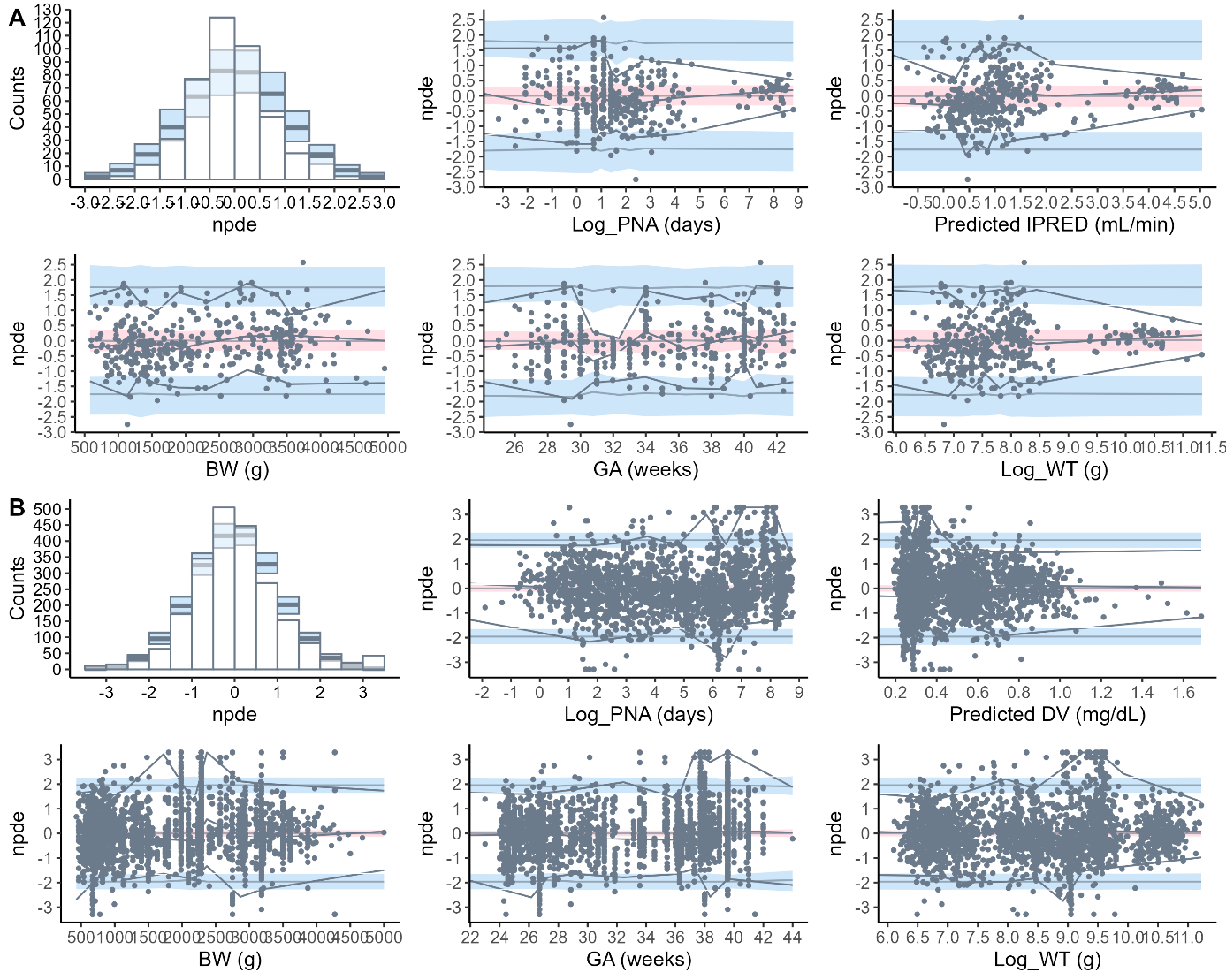


**Figure S1** Normalized Prediction Distribution Errors (NPDE) results for inulin clearance (A) and creatinine concentration (B). For each compound, subplots are respectively the distribution of NPDE shown as a histogram with blue area representing the 90% prediction interval under the theoretical N(0,1) distribution, the scatterplots of NPDE versus postnatal age (PNA) on a log scale, predicted creatinine concentration (CRT), birthweight (BW), gestational age (GA) and current weight (WT) on a log scale. Dots represents the NPDE computed for the dataset. The lines show the evolution of three empirical percentiles (2.5, 50 and 97.5) for the observed data (dark grey) compared to the model predictions (light grey). The pink band corresponds to the prediction interval for the median of the NPDE (50th percentile) and the blue bands the prediction intervals for the 2.5 and 97.5th percentiles.


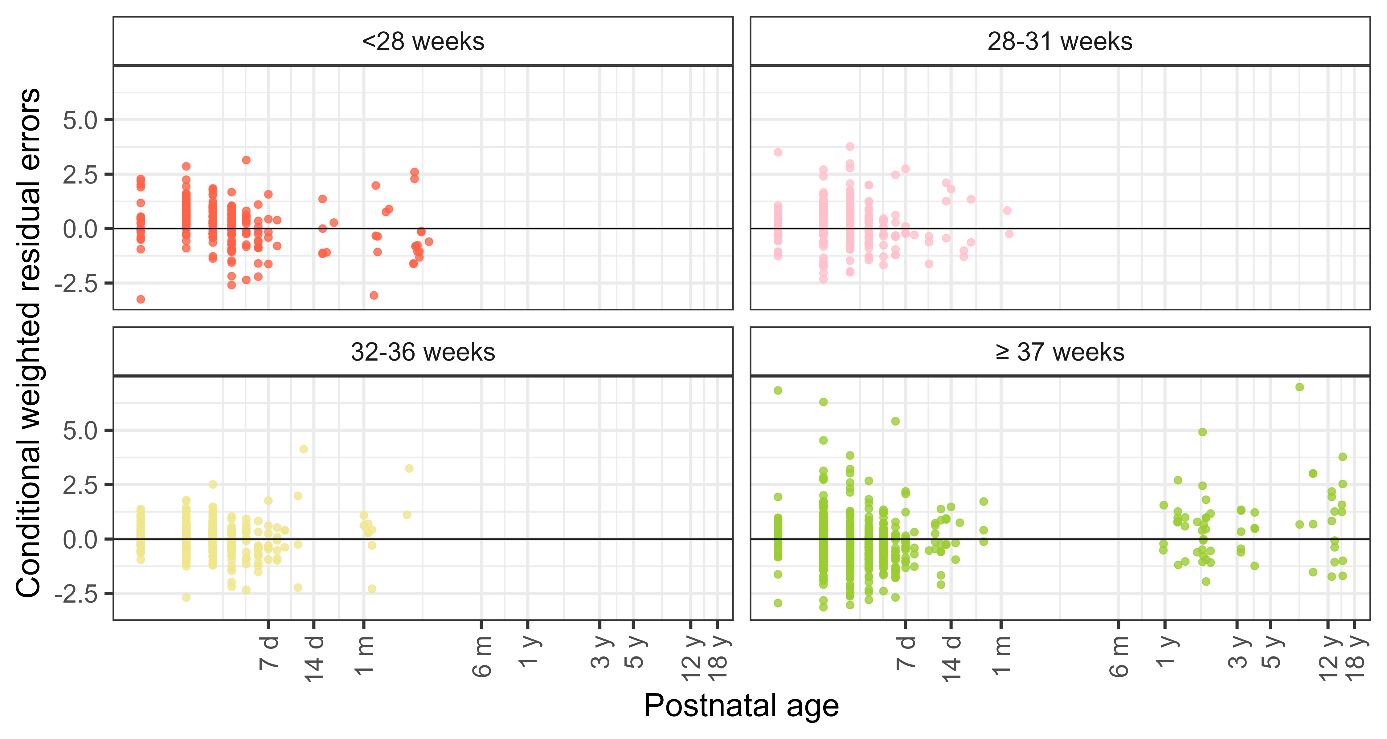


**Figure S2** Conditional weighted residuals *versus* postnatal age for the GFR-based model for gentamicin, split by four gestational age groups


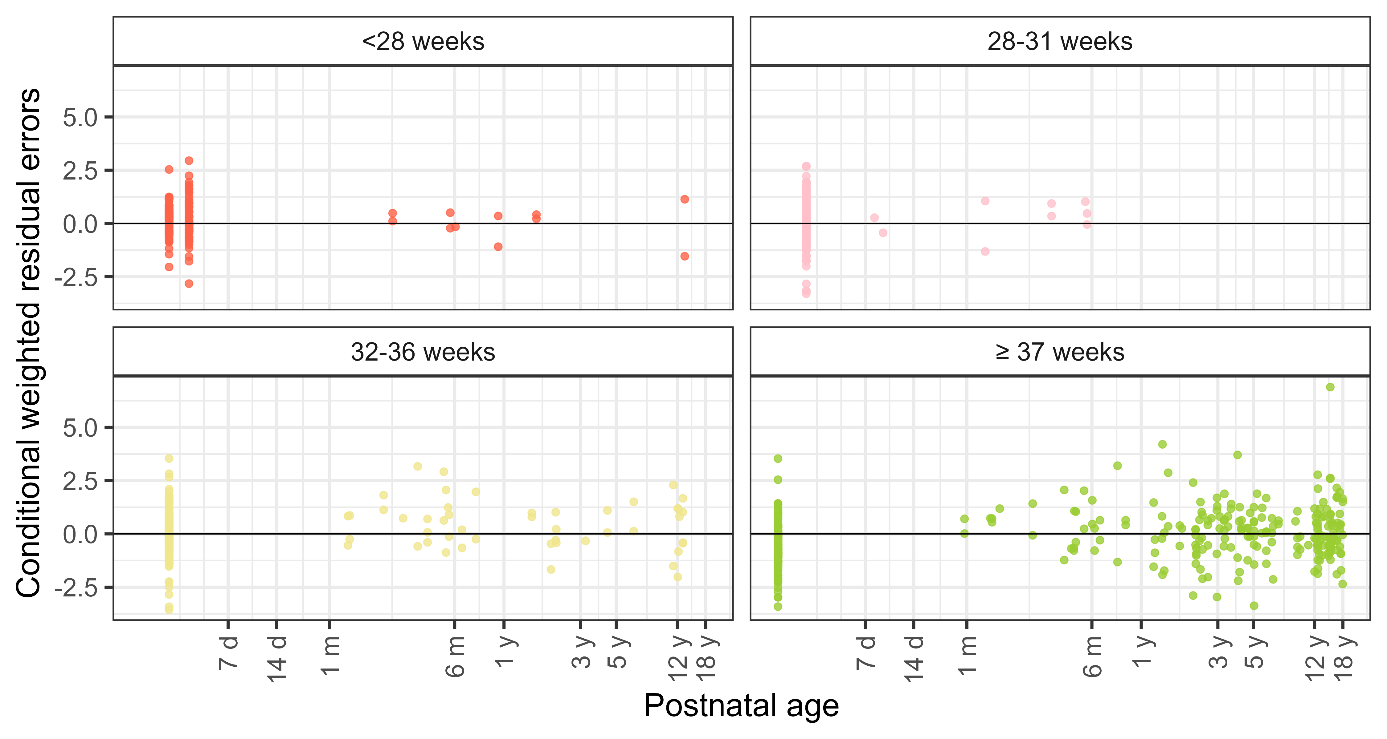


**Figure S3** Conditional weighted residuals *versus* postnatal age for the GFR-based model for tobramycin, split by four gestational age groups


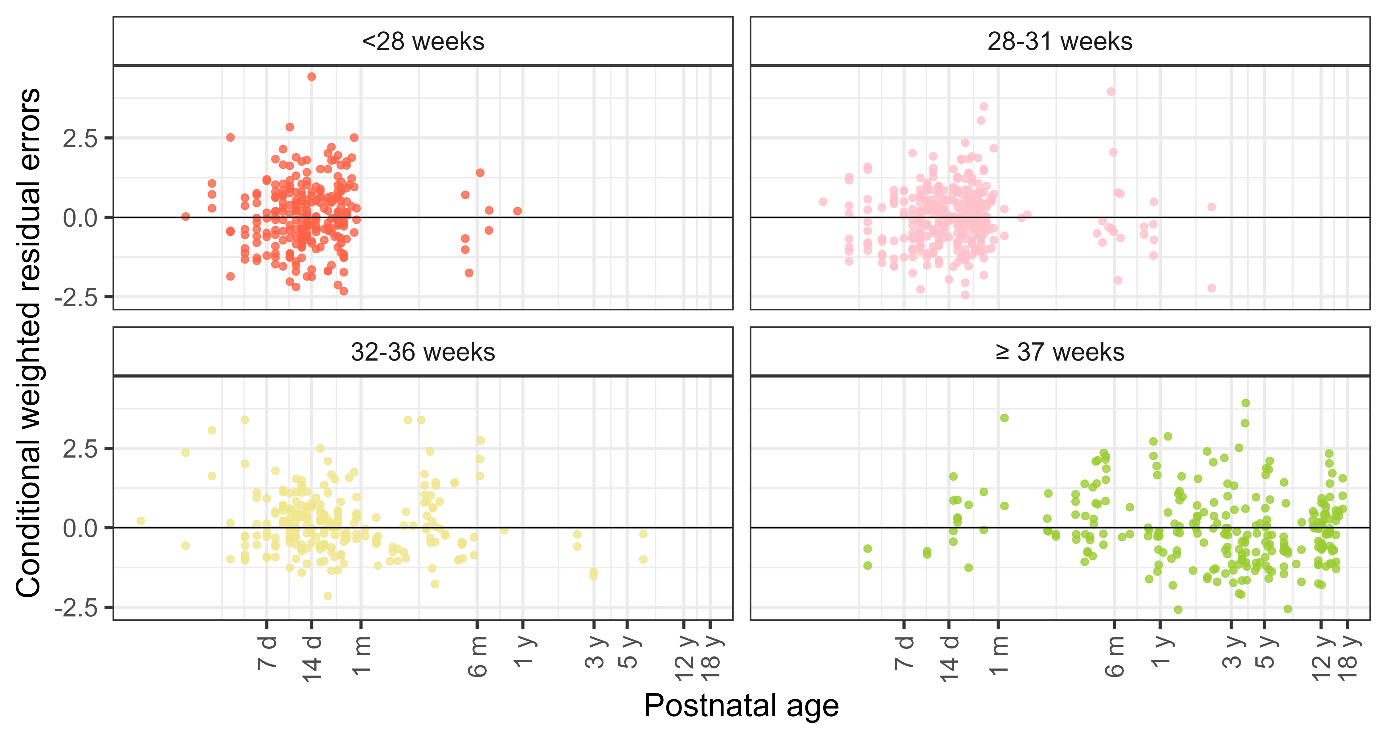


**Figure S4** Conditional weighted residuals *versus* postnatal age for the GFR-based model for vancomycin, split by four gestational age groups


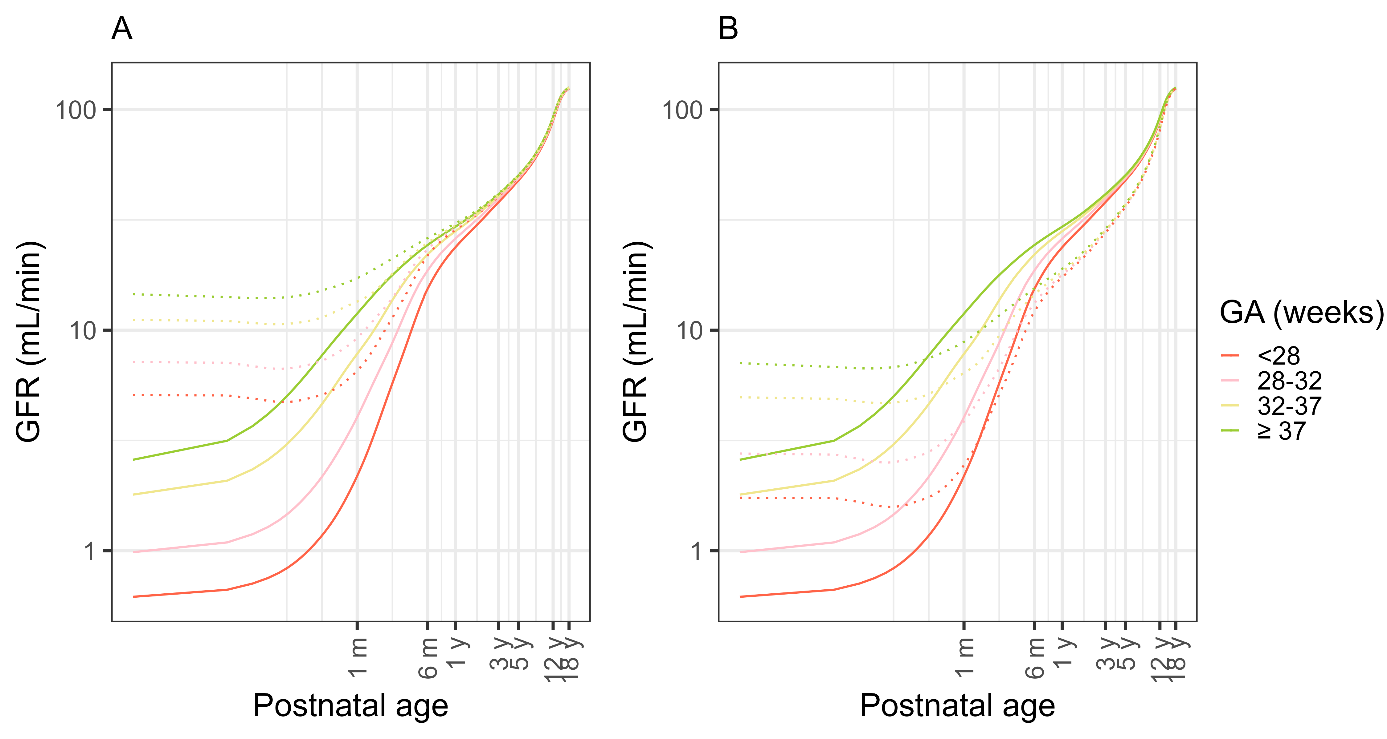


**Figure S5** Extrapolated GFR values (dashed lines) from 18 years to birth for different gestational age (GA) individuals using allometric scaling (A) and linear scaling (B) compared to the simulated GFR values (solid lines) using the GFR maturation model

**Ref:**

1. Landau-Crangle, E., et al., *Individualized Postnatal Growth Trajectories for Preterm Infants.* JPEN J Parenter Enteral Nutr, 2018. **42**(6): p. 1084-1092.

2. Paul, I.M., et al., *Weight Change Nomograms for the First Month After Birth.* Pediatrics, 2016. **138**(6).

3. Rodd, C., et al., *Extending World Health Organization weight-for-age reference curves to older children.* BMC Pediatrics, 2014. **14**(1): p. 32.

4. Fenton, T.R. and J.H. Kim, *A systematic review and meta-analysis to revise the Fenton growth chart for preterm infants.* BMC Pediatr, 2013. **13**: p. 59.

5. WHO. *WHO Weight-for-age growth charts*. Available from: <https://www.who.int/tools/child-growth-standards/standards/weight-for-age>.

6. Schwartz, G.J., L.P. Brion, and A. Spitzer, *The use of plasma creatinine concentration for estimating glomerular filtration rate in infants, children, and adolescents.* Pediatr Clin North Am, 1987. **34**(3): p. 571-90.

7. Brion, L.P., et al., *A simple estimate of glomerular filtration rate in low birth weight infants during the first year of life: noninvasive assessment of body composition and growth.* J Pediatr, 1986. **109**(4): p. 698-707.

8. Schwartz, G.J., et al., *New equations to estimate GFR in children with CKD.* J Am Soc Nephrol, 2009. **20**(3): p. 629-37.

9. Holness, J.L., et al., *Estimated glomerular filtration rate in children: adapting existing equations for a specific population.* Pediatr Nephrol, 2021. **36**(3): p. 669-683.

10. De Souza, V.C., et al., *Schwartz formula: is one k-coefficient adequate for all children?* PLoS One, 2012. **7**(12): p. e53439.

11. Pierce, C.B., et al., *Age- and sex-dependent clinical equations to estimate glomerular filtration rates in children and young adults with chronic kidney disease.* Kidney Int, 2021. **99**(4): p. 948-956.

12. Sherwin, C.M.T., et al., *Discrepancies between predicted and observed rates of intravenous gentamicin delivery for neonates.* Journal of Pharmacy and Pharmacology, 2010. **61**(4): p. 465-471.

13. Lopez, S.A., et al., *Extended-interval gentamicin: population pharmacokinetics in pediatric critical illness.* Pediatr Crit Care Med, 2010. **11**(2): p. 267-74.

14. de Hoog, M., et al., *Tobramycin population pharmacokinetics in neonates.* Clin Pharmacol Ther, 1997. **62**(4): p. 392-9.

15. Anderson, B.J., et al., *Vancomycin pharmacokinetics in preterm neonates and the prediction of adult clearance.* Br J Clin Pharmacol, 2007. **63**(1): p. 75-84.
